# Plans for New British Hospitals

**Published:** 1912-03-02

**Authors:** 


### HOSPITAL ARCHITECTURE AND CONSTRUCTION.

[Communications on this subject should be marked "Architecture" in the left-hand top corner of the envelope.]

### Plans for New British Hospitals.

### THE REFORMS URGENTLY NEEDED IN SELECTING PLANS.

We have often urged that in this country there is at present no adequate system for securing the selection by competition of the best plans submitted, much less of providing hospital committees with a guarantee that the new hospital when erected will be thoroughly up-to-date, modern and complete in every department. One practical reform which the architectural profession should insist upon, so far as the Royal Institute of British Architects is concerned, is the selection of a panel of experienced architects, competent on their merits, to act as assessors. Another is that the selection should never be left to the decision of one assessor, whoever he may be, for the reason that a modern hospital is many-sided and that no architect by himself has the knowledge to enable him to judge which out of many plans, so far as the administration and requirements of a modern hospital are concerned, is in fact the best. An architect may, if he is conscientious, knowledgeable, experienced and thorough, rightly be called upon to decide most points concerned with buildings, as such, which appertain to the professional work of an architect. For all the rest it is essential that there shall be associated with him a competent hospital expert who is admittedly an authority on the many technical and administrative matters which must materially affect a just and satisfactory decision as to the respective merits of a number of plans for a new hospital submitted in competition.

The causes of failure which have been apparent in the selection of plans in more than one recent competition in this country are every one of them due to absence of thoroughness on the part of the assessor, or to a want of thoroughness or of knowledge, or of practical hospital experience, or of a judicial mind to enable him first thoroughly to master and appraise all the conditions of competition and to disqualify every plan which does not comply with them. When a committee instructs the assessor to draw his conditions to include a hospital of a given number of beds not to exceed the maximum sum, such assessor is bound by his instructions, and should disqualify all competitors who submit plans the estimated cost of which exceeds the amount fixed for the guidance of every competitor.

A Grave Matter.

This question is a very grave one, and its gravity increases with every competition, so that we have determined to open our columns to a discussion on the points involved, in the hope that the necessary reforms may be speedily secured. Our columns are open to all who have practical points to raise, whatever their opinions may be, for it is essential to a successful issue in this matter that the truth shall be stated without fear or favour.

By way of illustration we publish this week criticisms on the awards in the most recent competition, in which no fewer than fifty-two sets of plans were submitted for adjudication and selection. As the immediate question affects primarily architects as a body, we have asked a Fellow of the Royal Institute of British Architects of great experience and position to study the whole of the plans submitted, to compare them with the plans selected, and to give our readers the benefit of the results he has arrived at. This special report is the first of the two articles which follow. The second communication has been sent to us spontaneously by another Fellow of the Royal Institute of British Architects, who has had considerable practical experience in hospital construction and hospital competitions of all kind.

### (1) Special Report on the East Sussex Hospital, Hastings.

In August last year invitations were issued to all architects practising in Great Britain to submit designs in competition for the proposed new hospital, and the fifty odd designs which have been sent in were on view during the week ending February 17 at the Drill Hall, Hastings. The conditions and instructions to competing architects were drawn up presumably by the Committee and the assessor jointly, as they are signed both by the secretary Mr. Rhodes, and the assessor, Mr. E. T. Hall.

The site on which the new buildings are to be erected is about four acres in extent, and while an excellent one in point of position and aspect, is a peculiarly difficult one to deal with on account of the levels. In shape it is roughly triangular, and along the whole of its south boundary abuts upon a public road. The general surface of the ground abutting on the road varies from about 7 feet at the east end to 20 feet at the west end above the level of the footpath, and the site itself has a fall of about 60 feet from west to east, and from north to south is approximately level. Crossing the site about midway in its length is a bank with a fall of about 7 feet.

A Preliminary Criticism.

The subsoil is sandstone rock. It will be seen, therefore, that the main difficulty lies in the treatment of the levels, both of buildings and approach roads, and, on economical grounds, the avoidance of excavation in the rock as much as possible. When it is further taken into consideration that the Committee fixed the sum of £35,000 (roughly £350 per bed) as the total cost of an up-to-date hospital, the thought naturally occurs to us that neither the Committee nor the assessor realised at all the difficulties which competitors were asked to solve. The amount fixed as a maximum is inadequate enough if the site had been a dead level, but it is hopelessly insufficient for a building on such a site as this.

The hospital is to be for 102 beds, with provision for future extension. No information, however, is given as to the size of this future extension; in fact, in answer to a question put by a competitor, the assessor says: "Design only for specified buildings, with room for future extension." A more inept reply could hardly have been made. It should have been quite impossible to decide upon some figure for the ultimate number of beds on the site, and clear directions ought to have been given on this point.

The allocation of wards is as follows:—

| | |
|---|---|
| 1—men's medical ward, 12 beds, side ward, 2 beds ... | 14 |
| 2—men's surgical ward, 14 beds, side ward, 4 and 2 beds ... | 20 |
| 3—men's isolation ward, 2 beds ... | 2 |
| 4—men's eye ward, 3 beds... | 3 |
| 5—women's surgical ward, 14 beds, side wards, 4 and 2 beds ... | 20 |
| 6—Women's medical ward, 14 beds, side ward, 2 beds | 16 |
| 7—women's isolation ward, 2 beds ... | 2 |
| 8—women's eye ward, 3 beds ... | 3 |
| 9—gynæcological ward, 10 beds ... | 10 |
| 10—children's ward, 12 beds ... | 12 |
| Total ... | 102 |

The limit of cubic space (the far more important question of floor area is not mentioned) is fixed at 1,400 for all wards except those for children, which is fixed at 1,000 feet. The schedule of instructions is a long one, and while in some instances it goes into minute details, in others it is drawn so carelessly as to give rise to an inordinately long list of questions by competitors in order to elucidate the meaning. Points which ought to have been settled definitely have been left open, while others on which competitors should have been allowed a free hand are rigidly fixed. For example, information is sought as to the numbers respectively of sisters, nurses, and probationers; the requirements including separate sitting-rooms for each of these classes. The only information vouchsafed is that there would be about seven sisters, and that a common room for nurses and probationers would be accepted. Surely it should not be beyond the power of the authorities to say exactly how many sisters would be required!

A curious requirement in the schedule is for "patients' box-rooms," and on a competitor asking for what purpose these are required the answer is given, "lumber-rooms, etc.!" The question is asked whether the nurses' w.c. in the ward block may be in the sanitary tower, and the answer is, "not desirable." Why not desirable in the name of common-sense? Appended to the schedule is a list of recommendations by the committee, most of which would have been better omitted altogether. The impression left in our mind after reading the schedule and the questions by competitors with the assessor's replies is that the whole scheme has been carelessly drawn up and shows in many instances a lamentable lack of knowledge on the part of their adviser.

When we come to examine the plans we are at a complete loss to understand how any assessor with any knowledge of hospital planning could have arrived at such a decision as has been given in this case. Of the three premiated plans Nos. 1 and 2 ought not to have been placed in the first half-dozen and No. 3 is superior to the other two in almost every point. The designs as a whole are, it must be confessed, disappointing; comparatively few of the competitors have made any attempt to construct their roads economically, and many have been led into the error of multiplying entrances to get over the difficulties of gradients.

### The First Design.

In the design placed first the road leading to the mortuary has a slope of 1 in 3 as calculated from the figured levels, although the elevation shows a nearly level approach. The road to the out-patient department has a slope of 1 in 4.7, and that to the main administration block a slope of 1 in 5. Needless to say that such gradients are wholly impossible for wheeled traffic and quite steep for foot traffic. These points alone ought to have disqualified the design.

Turning to the plans we find the out-patient department and the dispensary placed at a level sixteen feet below the ward corridor; the only connection between the two being by two long flights of stairs within a covered way. All medicines, etc., therefore served from the dispensary for the wards would have to be carried up these stairs as there is no lift and no possibility of arranging one.

The ward pavilions are so planned that when the future extension is carried out the eye wards and two of the two-bed wards must be destroyed. The eye wards instead of being grouped together with a common operation-room are placed one with the male, the other with the female wards, and two operation theatres are provided. On the ground floor no sanitary provision is made for the eye wards and the two small wards; the only offices being at the extreme end of the large wards. The same remark applies to the first floor, where are wards for four and two patients with no sanitary accommodation, except that at the south end of large ward. Influenced we presume by the absurd answer to the question as to the position of nurses' w.c., the author has arranged these offices off the main corridor—about the most inappropriate position he could have chosen.

### Remarks on the Unsuccessful.

In many respects the design placed second is superior to that placed first, but here again the difficulty of levels is not dealt with in a satisfactory way. The design placed third is, as we have said, distinctly superior to the other two. The planning of the roads is carefully worked out with due regard to the levels of the site. In point of general planning the design shows much greater knowledge of the subject than either Nos. 1 or 2.

The out-patient department is not well arranged, the author having apparently thought more of getting a good hall for concerts and large meetings than of the convenient working of the department. This is perhaps the weakest part of the plan, which is on the whole a good one.

### (2) Note on East Sussex Hospital, Hastings.

The competition for the East Sussex Hospital has proved to be a difficult problem for the competitors, few of whom seem to have solved the perplexities of the site, if, indeed, they have realised what it involved. The assessor must have also been embarrassed, as the selected design ignores all difficulties and solution of levels.

The site, ideal in itself, is on the heights with a bird's-eye prospect of the sea, situated in a pine wood. The only approach is from the main road to Ore, which has a steep gradient, and the frontage is about 700 feet long on a perpendicular bank of stone and loam. This bank is about eight feet above the path at the lower end and over twenty feet at the upper, the site falling in three terraces. The problem was, therefore, to get from a roadway of steep gradient to the top of the site, and, secondly, when there to conquer a gradient steeper than that to the roadway beneath, dropping together forty feet. The third problem was to place the buildings in the most economical way with serviceable and equal levels and to procure easy access with a minimum of roadmaking on an expensive site.

The beautiful site by its inaccessibility has long been on the market as unsuitable for development. Naturally, it would do for a hospital, and it would have done provided that a suitable design had been selected. That this has been the case we have grave reason to doubt. The assessor's conditions show a lack of knowledge both of the site and the application of the conditions thereto; it being assumed that the site was level. From an examination of the selected design it is clear that the difficulties have been ignored, and this has not been recognised by the assessor. The cost was stipulated as being an essential in the selection, £35,000 being the sum that the committee would have available. The selected design is about £7,000 above the sum fixed as essential in the selection. I believe that with care a much lower figure might be reached.

